# Time of proto-Earth reservoir formation and volatile element depletion from ^53^Mn-^53^Cr chronometry

**DOI:** 10.1126/sciadv.adw1280

**Published:** 2025-08-01

**Authors:** Pascal M. Kruttasch, Klaus Mezger

**Affiliations:** Institut für Geologie, Universität Bern, 3012 Bern, Switzerland.

## Abstract

The ^53^Mn-^53^Cr chronometry of Solar System materials constrains the early chemical evolution of the protoplanetary disk, which is critical for planet formation. Mn/Cr ratios in carbonaceous chondrites and the bulk silicate Earth indicate that meteorite parent bodies and Earth have variable depletions in volatile elements compared to the bulk Solar composition. This depletion is a consequence of the local temperature decreasing as a function of heliocentric distance before planetesimal accretion. Back-tracking the present-day ε^53^Cr composition of the hypothetical proto-Earth fraction shows that the cessation of Mn-Cr fractionation from the bulk Solar composition occurred no later than ~3 Ma after CAI formation, similar to disk regions of carbonaceous chondrites at greater heliocentric distances. The timing of limited solid-gas interaction due to the dissipation of gas from the protoplanetary disk caused the cessation of Mn-Cr fractionation and provides a lower limit on its lifetime.

## Teaser

Reservoirs of proto-Earth and carbonaceous chondrites lost their volatile element budget in the first ~3 Ma of the Solar System.

## INTRODUCTION

Evaporation and condensation of solids in the protoplanetary disk are fundamental processes in the evolution of the Solar System that shaped the volatile element inventory of planetary building blocks. Volatility-controlled fractionation processes and their timescales in different regions of the protoplanetary disk are key to understanding the evolution of the protoplanetary disk and, ultimately, the formation of habitable planets, such as Earth. Evaporation and condensation are thought to have occurred throughout the protoplanetary disk stage from nebula gas until its dissipation by photo-evaporation, viscous accretion, and planetesimal formation [e.g., (*1*)]. Astronomical observations suggest that the dissipation of gas and the associated transition from protoplanetary to debris disks (through a transitional disk stage) occurs in the first ~3 to 5 million years (Ma) of a disk’s lifetime (*2*–*4*). However, less is known about the disk evolution of our Solar System and the formation of planetary reservoirs involving volatile element fractionation processes. The evolution of the short-lived ^53^Mn-^53^Cr system [*t*_1/2_ = 3.80 ± 0.23 Ma (*5*)] in planetary materials provides precise age constraints for these processes. Manganese and Cr are moderately volatile elements with 50% condensation temperatures of $T_{c}^{\text{Mn}}$ = 1123 K and $T_{c}^{\text{Cr}}$ = 1291 K at 10^−4^ bar (*6*), which can result in relatively large Mn-Cr fractionation in different planetary building blocks. This makes the ^53^Mn-^53^Cr chronometer suitable for dating chemical differentiation in the cooling solar nebula gas (*6*, *7*) and evaporation from silicate melt (*8*). In addition, Mn and Cr fractionate in silicate-metal melt systems as a function of S concentration due to their different chemical affinities [e.g., (*9*, *10*)].

This study models the ε^53^Cr evolution of the reservoirs of proto-Earth (PE) and carbonaceous chondrites (CCs) by tracing their present-day ε^53^Cr backward through time to constrain the time of reservoir formation and volatile element fractionation events in the evolving protoplanetary disk. It is assumed that ^53^Mn and ^53^Cr were uniformly distributed at the beginning of Solar System formation (*5*, *11*), and the initial ^55^Mn/^52^Cr ratio of the bulk Solar System (as represented by CI chondrite) was ^55^Mn/^52^Cr_CI_ = 0.84 (*12*). This resulted in a substantial ingrowth of ~0.5 ε^53^Cr units in the first ~10 million years of the Solar System, which is much higher than the current analytical precision of typically less than 0.1 ε^53^Cr (2SE). The present-day bulk silicate Earth (BSE) and CCs (except CI) have ε^53^Cr compositions lower than CI [e.g., Δε^53^Cr_BSE-CI_ = −0.24 ± 0.02 (*12*)]. This peculiarity indicates that Mn-Cr fractionation of these reservoirs occurred early from the bulk Solar composition, resulting in different pathways of ε^53^Cr evolution through time. On the basis of the Mn/Cr ratio of today’s BSE (*13*), it is expected that the PE had a low Mn/Cr ratio compared to the bulk Solar System value as recorded in the Sun and CI chondrite (*7*). The generally lower abundances of volatile elements in the present-day BSE indicate that Earth, or its precursor materials, lost the major inventory of volatile elements early during their evolution [e.g., (*14*)]. Chondrites, which are mostly undifferentiated material from the early stages of the evolving Solar System and possibly the precursors of the terrestrial planets, exhibit different extents of volatile element depletion [e.g., (*14*, *15*)]. A comparison of element and isotope abundances of the Earth with meteoritic materials indicates that Earth itself cannot be the product of a single meteorite group [e.g., (*16*)]. The distinct and variable element abundances of the present-day BSE (*13*) suggest that Earth consists of at least three chemically and isotopically distinct components. These components likely mixed during the Moon-forming impact, ≲70 Ma after the formation of the Solar System and thereafter (referred to as “late-veneer”) (*17*, *18*). The completion of Earth’s core formation was contemporaneous with the Moon-forming impact [e.g., (*19*)]. Today’s element abundances of the BSE and isotope constraints strongly suggest that the bulk Earth comprises ~90% PE, ~10% Theia, and ~0.4% late veneer material, where Theia and the late veneer material had high volatile element contents approaching those of CI chondrite, while the PE was strongly depleted in volatile elements (*14*, *18*, *20*, *21*). Other models suggest that Earth formed from ~60% PE and ~40% Theia [e.g., (*22*)], where Theia had a composition similar to enstatite chondrites (*23*–*26*), i.e., similar to PE.

To obtain age information on the time of Mn-Cr fractionation events, as recorded in the PE and CCs relative to the bulk Solar System, this study constrains (i) the bulk Solar System ^53^Mn/^55^Mn and ε^53^Cr at any absolute time; (ii) the present-day ^55^Mn/^52^Cr and ε^53^Cr of the hypothetical PE, proto-Earth mantle (PEM), and proto-Earth core (PEC); and (iii) traces back the ε^53^Cr composition of the PE and CCs reservoirs through time. The time of PE reservoir formation is constrained assuming accretion of Earth building blocks via oligarchic growth of Moon- to Mars-sized planetary embryos (*27*) for three different mixing scenarios between PEM and Theia that result in the present-day ^55^Mn/^52^Cr and ε^53^Cr of the BSE. Model I assumes that PEM and Theia had identical compositions (*23*–*26*, *28*), thus independent of the relative mass fraction between PEM and Theia. Model II assumed that 90% of the material is PEM and 10% Theia (± 5%), where Theia is CI chondrite-like (*18*, *20*, *21*, *29*, *30*). Model III assumes that 60% of the material is PEM and 40% Theia (± 5%), with Theia being CI chondrite-like.

## RESULTS

**Fig. 1.**
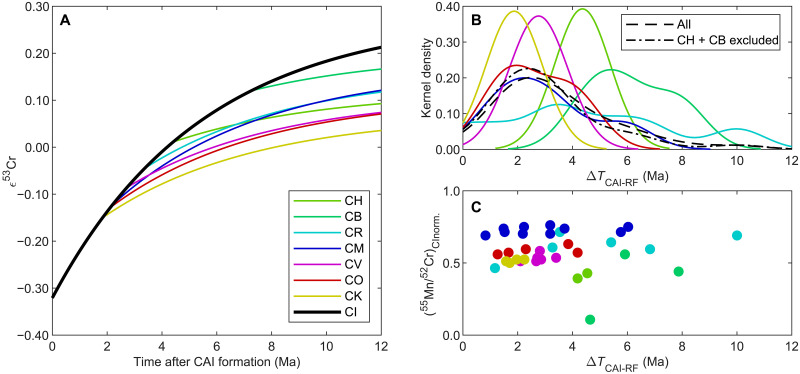
ε^53^Cr evolution of CCs and their intersection ages (model ages) with the bulk Solar System. (**A**) Average ε^53^Cr evolution curves of CC groups. (**B**) Kernel density distribution of reservoir formation (RF) ages of CC groups using individual CC meteorites shown in (C). (**C**) CI normalized ^55^Mn/^52^Cr ratios of individual chondrite samples as a function of RF ages. The ages are relative to CAI formation at 4568.54 Ma. Carbonaceous chondrite data are from Zhu *et al.* (*12*). The meteorite Maribo (CM2) is excluded due to an exceptionally high present-day ε^53^Cr of 0.29 ± 0.04 (*12*).

Relative to the bulk Solar System ^55^Mn/^52^Cr_CI_ [= 0.84 (*12*)], CCs are depleted in volatile elements to varying degrees and thus have lower Mn/Cr ratios (*12*). By back-calculating from the present-day ε^53^Cr composition using ^55^Mn/^52^Cr of CCs and a single-stage and instantaneous fractionation event, their reservoir formations (in conjunction with Mn-Cr fractionation from the bulk Solar System) are constrained by the intersections of their ε^53^Cr evolution curves with the bulk Solar System ε^53^Cr evolution curve (Fig. 1). Using the parameters derived for the ^53^Mn-^53^Cr systematics, single-stage model ages are derived for the fractionation of Mn from Cr for different Solar System materials. This results in reservoir formation ages of CCs (except CB and CH) from $4565.2_{-4.6}^{+6.0}$ to $4566.7_{-0.7}^{+0.8}$ Ma, i.e., $1.8_{-0.7}^{+0.8}$ to $3.4_{-4.6}^{+6.0}$ Ma after the formation of calcium-aluminium-rich inclusions (CAIs) (Table 1), that coincide with the time interval of chondrule formation (*31*–*34*). CB and CH chondrites have distinctly later reservoir formation ages from $4561.3_{-0.6}^{+0.7}$ to $4564.1_{-0.9}^{+1.1}$ Ma (i.e., $4.4_{-0.9}^{+1.1}$ to $7.3_{-0.6}^{+0.7}$ Ma after CAIs), consistent with some late formation ages of CB chondrules [e.g., (*35*)].

**Table 1.** Absolute and relative ages of the intersections of ε^53^Cr evolution curves of the reservoirs with the bulk Solar System.

	Absolute age (Ma)	Δ*T* after CAI (Ma)*	
CH	4564.1	4.4	+1.1/−0.9
CB	4561.3	7.3	+0.7/−0.6
CR	4565.2	3.4	+6.0/−4.6
CM	4566.3	2.2	+9.3/−3.3
CV	4565.8	2.7	+1.5/−1.2
CO	4566.4	2.1	+2.9/−1.9
CK	4566.7	1.8	+0.8/−0.7

*Δ*T* is relative to CAI formation at 4568.54 Ma. Uncertainties are 2SD.

The formation age of the PE reservoir requires constraints on its hypothetical present-day ε^53^Cr and ^55^Mn/^52^Cr. Because the present-day BSE has lower ^55^Mn/^52^Cr and ε^53^Cr [^55^Mn/^52^Cr_BSE_ = 0.475 (*13*); ε^53^Cr_BSE_ = 0.04 ± 0.02 (*12*)] than CI chondrite [^55^Mn/^52^Cr_CI_ = 0.84 ± 0.04 and ε^53^Cr_CI_ = 0.28 ± 0.01 (*12*)], the present-day PEM fraction must have ^55^Mn/^52^Cr and ε^53^Cr that are equal to or lower than those of the BSE (Table 2). Metal-silicate partition coefficients of Mn and Cr indicate that $D_{\text{Mn}/\text{Cr}}^{m-s}$ is <0.16 (*9*, *10*). Consequently, ^55^Mn/^52^Cr of PE and PEC is systematically lower than PEM (Table 2). The ε^53^Cr value of the hypothetical present-day PEC is constrained between the Solar System initial (ε^53^Cr = −0.32 ± 0.06) and the hypothetical present-day ε^53^Cr of PEM. This results by mass balance in a hypothetical present-day ε^53^Cr composition of PE between PEM and PEC (Table 2).

**Table 2.** ^55^Mn/^52^Cr and ε^53^Cr of PEM, PEC, and PE for three different scenarios (Fig. 2), where Theia is similar to BSE or CI chondrite, and contributes 10 to 40% of mass to the present-day Earth. The mass fraction of the Earth core (Χ_PEC_ and χ_PEC_) and the Mn/Cr ratio of metal-silicate partition coefficients are the same in all three models.

	Model I	Model II	Model III
	PEM = Theia = BSE	90% PEM:10% Theia	60% PEM:40% Theia
		(±5%)	(±5%)
^55^Mn/^52^Cr_Theia_	0.475	0.84	0.84
ε^53^Cr_Theia_	0.02	0.28	0.28
Θ_Mn/Cr_	0	0.8–0.9	1.2–1.4
Χ_PEC_ and χ_PEC_	0.325		
$D_{\text{Mn}/\text{Cr}}^{m-s}$	0–0.16		
^55^Mn/^52^Cr_PEM_	0.475	0.411–0.456	0.177 – 0.279
^55^Mn/^52^Cr_PEC_	0.000–0.076	0.000–0.073	0.000 – 0.045
^55^Mn/^52^Cr_PE_	0.321–0.345	0.277–0.331	0.119 – 0.203
ε^53^Cr_PEM_	0.02–0.06	−0.03–0.05	−0.19 – −0.06
ε^53^Cr_PEC_	−0.32–0.06	−0.32–0.05	−0.32 – −0.06
ε^53^Cr_PE_	−0.09–0.06	−0.12–0.05	−0.23 – −0.06

**Fig. 2.**
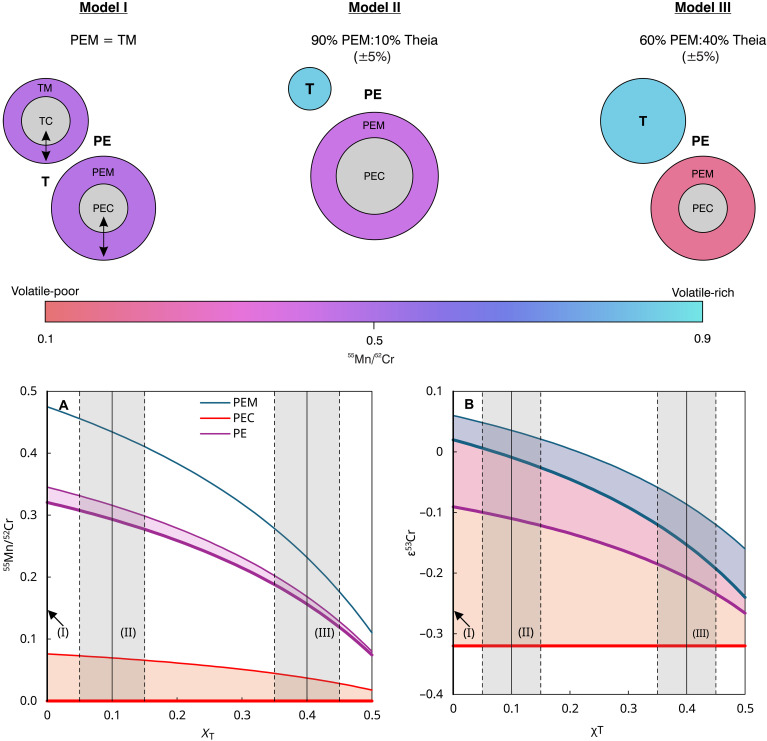
Three hypothetical mixing scenarios between the PE and Theia. Mixing of Mn/Cr between PEM and Theia (mantle) in all cases results in the present-day composition of the BSE. Model I assumes mixing of PEM and Theia with identical Mn/Cr (similar to BSE), independent of size; model II assumes mixing of 90% PEM:10% Theia (± 5%), where Theia’s Mn/Cr is CI chondrite-like; model III assumes mixing of 60% PEM:40% Theia (± 5%), where Theia’s Mn/Cr is CI chondrite-like. Note that in models II and III (Mn/Cr of Theia similar to CI chondrite), Theia was probably too oxidized to form a core. In contrast, under more reduced conditions (model I), Theia likely underwent core formation resulting in Theia mantle (TM) and Theia core (TC). (**A**) ^55^Mn/^52^Cr as a function of the bulk mass fraction of Theia (Χ_T_), and (**B**) ε^53^Cr as a function of Cr mass fraction of Theia (χ_T_) for PEM, PEC, and PE, respectively. The mass fractions of Theia for models II and III are shown as gray areas or on the ordinate for model I.

**Fig. 3.**
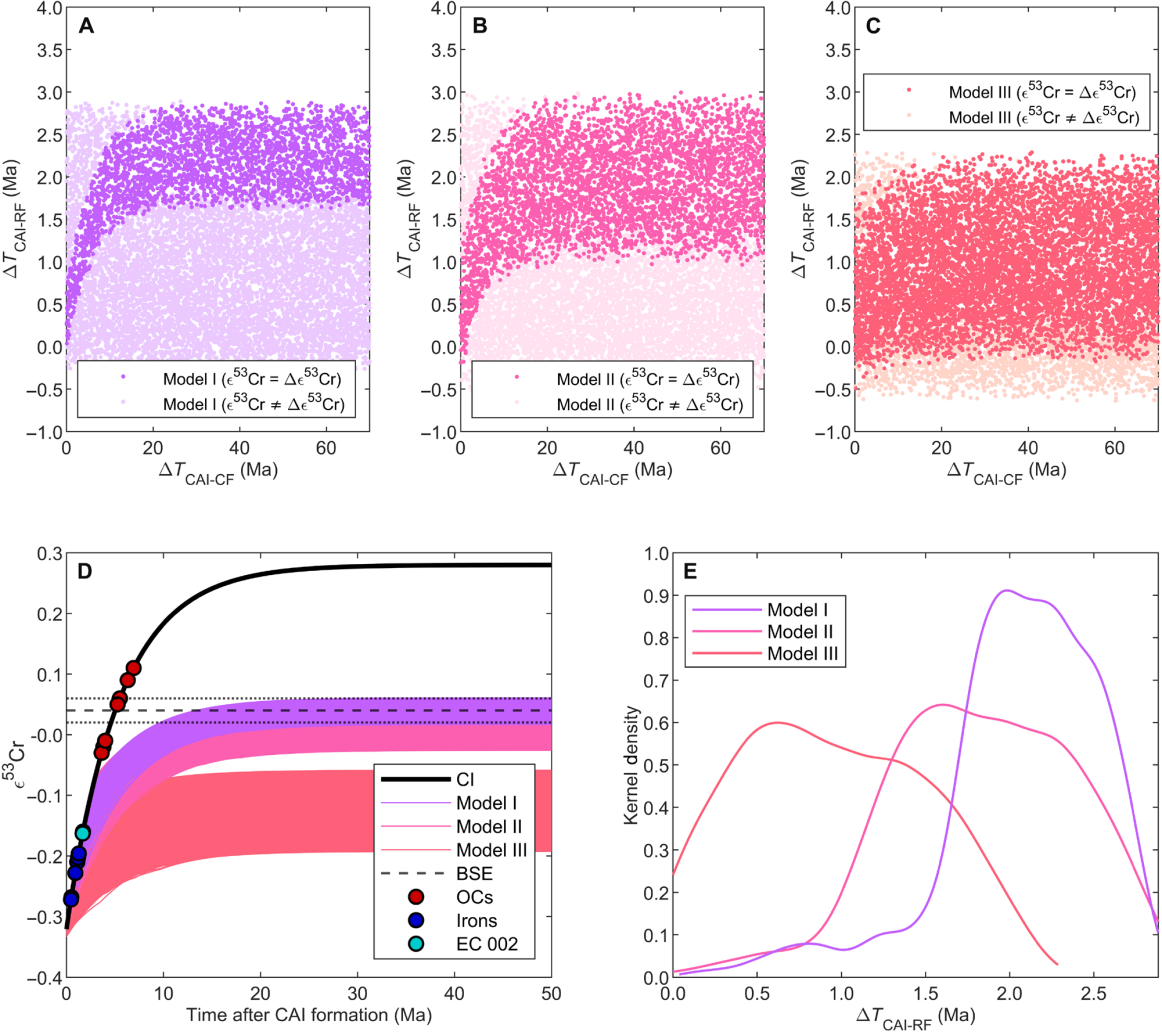
ε^53^Cr evolution of PE-PEM for the three different mixing scenarios. The ε^53^Cr evolution is modeled using 10,000 Monte Carlo simulations with variable ^55^Mn/^52^Cr of PE and PEM and present-day ε^53^Cr of PE, and Mn-Cr fractionation of PE into PEM at time *t*_1_ (core formation) between 0 and 70 Ma after CAI formation. (**A** to **C**) Reservoir formation (RF; PE-CI intersection) ages as a function of core formation (CF; PE-PEM fractionation) ages for the three different mixing models [(A) model I; (B) model II; (C) model III). Dark colors indicate simulations between ε^53^Cr evolution of PE-PEM resulting in the present-day ε^53^Cr of PEM; light colors indicate simulations that do not reproduce the present-day ε^53^Cr of PEM. (**D**) ε^53^Cr time evolution of filtered Monte Carlo simulations of PE-PEM resulting in the constrained range of the present-day ε^53^Cr of PEM. The ε^53^Cr compositions of chromite grains (with ^55^Mn/^52^Cr near zero) from iron meteorites, ordinary chondrites, and Erg Chech 002 (*36*, *38*, *39*) are plotted on the bulk Solar System evolution curve for comparison. (**E**) Kernel density distribution of PE reservoir formation ages resulting in present-day ε^53^Cr of PEM. Δ*T*_CAI-RF_ and Δ*T*_CAI-CF_ are the times of RF and CF relative to CAI formation at 4568.54 Ma.

Modeling the ε^53^Cr evolution of PE-PEM through time using 10,000 Monte Carlo simulations for the three mixing scenarios (Fig. 2) with variable ^55^Mn/^52^Cr of PE and PEM and ε^53^Cr of PE (Table 2) and variable times of core formation between 0 and 70 Ma results in PE intersections with the bulk Solar System evolution curve between ε^53^Cr = −0.32 and ε^53^Cr = −0.07. This constrains Mn-Cr fractionation in the PE reservoir to no later than 3 Ma after the formation of CAI (Fig. 3). The kernel density distributions of PE reservoir formation ages of model I and II suggest that volatile element depletion occurred no later than 3 Ma with maximum likelihoods at 1.7 and 2.0 Ma after the formation of the Solar System, respectively. In contrast, the kernel density distribution of PE reservoir formation ages of model III peaks at 0.5 Ma and approaches zero after the first 2 Ma of the Solar System (Fig. 3E).

## DISCUSSION

The ε^53^Cr and ^55^Mn/^52^Cr of different CC groups (excluding CB and CH) yield single-stage model ages for volatile element depletion ranging from $1.8_{-0.7}^{+0.8}$ to $3.4_{-4.6}^{+6.0}$ Ma after CAI formation based on their intersection with the bulk Solar System (CI) evolution curve (Fig. 1A). These model ages represent average ages for each CC group containing chondrules, refractory inclusions, and a fine-grained matrix, which potentially formed at different times and been mixed later. The last Mn-Cr fractionation event in chondrules and matrix controls these model ages, as the contribution of Mn and Cr in refractory inclusions to the bulk rock composition is negligible. Regardless of the extent of mixing, the single-stage model ages provide mean ages for an ongoing depletion that may have affected different regions at different times and lasted for ca. 1.5 Ma. This time interval of Mn-Cr fractionation coincides with the period of chondrule formation [e.g., (*31*–*34*)] and is at the lower end of the formation and differentiation ages of ordinary chondrite parent bodies [e.g., (*36*)]. In contrast, CH and CB chondrites show younger reservoir formation ages between $4.4_{-0.9}^{+1.1}$ to $7.3_{-0.6}^{+0.7}$ Ma after CAI formation. In these two groups, chondrules likely formed from impact-induced vapor plumes, which is in stark contrast to chondrule formation processes that produced the chondrules of other chondrite groups (i.e., enstatite chondrites, ordinary chondrites, and CCs) that formed by brief heating events of nebular dust accumulations (*37*). Hence, the distinct Mn-Cr fractionation ages of CCs suggest that chondrite components formed episodically at different times and by different processes in the protoplanetary disk.

The average ε^53^Cr model age of CCs, as constrained by the intersection of CCs and the bulk Solar System, peaks at ~2.4 Ma after the formation of CAIs (Fig. 1B). This peak may reflect the maximum of Mn-Cr fractionation events, which occurred after the formation of the first differentiated meteorite parent bodies [i.e., magmatic iron meteorite parent bodies and the parent body of Erg Chech 002 (*38*, *39*)]. The occurrence of early-formed magmatic iron meteorite parent bodies, which bear isotope signatures from the inner [noncarbonaceous chondrite (NC) reservoir] and outer (CC reservoir) Solar System [e.g., (*40*)], suggests disk-wide early accretion and differentiation in distinct protoplanetary disk regions. The magmatic differentiation of early-formed planetary bodies requires a high abundance of the short-lived radioisotope ^26^Al, which likely was the primary heat source in the earliest planetary bodies. Hence, the peak of Mn-Cr fractionation at ~2.4 Ma after CAI formation relates to the declining abundance of ^26^Al as a heat source (with a half-life of ^26^Al of 0.717 Ma; National Nuclear Data Center NuDat v2.7, 2018), which limited internal heating and led to the formation of undifferentiated parent bodies at different locations in the disk. To prevent global melting of planetary bodies, the heat supply from ^26^Al decay must have been less than 1.6 kJ/g (*41*), which was achieved after ~1.5 Ma of Solar System evolution, assuming an initial ^26^Al/^27^Al of 5.23 × 10^−5^ and volatile-rich dust containing about 0.9 weight % (wt %) Al [similar to CI chondrite (*42*)]. The abundance of siderophile volatile elements (e.g., S) in some early-crystallized iron meteorites suggests that some initial liquid metal cores, representative of their parent body inventory, were volatile-rich [e.g., (*43*)]. This suggests that volatile-rich matter condensed and may have been recycled early on. The presence of an initially volatile-rich inventory in early-formed meteorite parent bodies is also supported by the element abundances in some achondrites, such as ureilites, eucrites, and other meteorites related to vestoids (e.g., Northwest Africa 12217), which suggests that their parent bodies accreted in the first ~1.5 Ma of the Solar System [e.g., (*44*–*46*)].

In contrast to volatile-rich materials such as CI, the PE must have been strongly volatile-depleted, as evident from the abundance of lithophile volatile elements in the BSE (*20*, *21*). The ^55^Mn/^52^Cr depletion of PE relative to the solar ratio suggests that either PE lost large amounts of volatile elements during its evolution or the material that formed PE was already largely depleted, reflecting volatile element fractionation processes in the solar nebula. In contrast to PE, many studies proposed that Theia had near solar abundances of refractory to at least the moderately volatile elements (*18*, *20*, *21*, *29*, *30*), suggesting that it did not experience extensive volatile element depletion.

The distinctively higher Mn/Cr ratios of CCs compared to the PE reservoir may be the consequence of the local temperature in the protoplanetary disk, which decreased as a function of heliocentric distance. This temperature gradient in the disk may have controlled the distinct volatile element inventories of the terrestrial Solar System planets—Mercury, Venus, and Mars—and asteroids such as 4Vesta. While Mars is richer in volatile elements than PE [^55^Mn/^52^Cr_Mars_ = 0.59 (*47*)], the inner Solar System planets Venus and Mercury are largely devoid of volatile elements, with an estimated ^55^Mn/^52^Cr of ~0.14 and ~0.03, respectively (*48*). Although asteroid 4Vesta, as sampled by howardite-eucrite-diogenite (HED) meteorites, exhibit relatively high Mn/Cr (*49*), it may have lost more volatile elements by parent body processes (*50*). This gross volatile depletion in the terrestrial planets as a function of heliocentric distance indicates that Theia, which in the case of models II and III, is richer in volatile elements than PE, formed with higher than or equal to Martian ^55^Mn/^52^Cr ratio at a correspondingly greater distance from the Sun. However, other studies (*23*–*26*, *28*) propose that PE and Theia had nearly identical chemical and isotope compositions similar to those of enstatite meteorites.

Model I to III (Fig. 2) cover nearly the spectrum of proposed mixing scenarios involving differences in mass fraction and chemical and isotope composition of Theia and PE. This ranges from large chemical differences (high Θ_Mn/Cr_) between PE and Theia [model III: 60% PEM and 40% Theia (±5%), where Theia is similar to CI-chondrite] to PE and Theia having identical compositions (model I; Θ_Mn/Cr_ = 0). Model II [90% PEM and 10% Theia (±5%), where Theia is similar to CI-chondrite] is intermediate to these end-member scenarios and favored to explain most of the chemical and isotope signatures of the present-day BSE (*14*, *18*, *20*, *21*, *30*, *51*, *52*).

Monte Carlo simulations that assume for all three scenarios that the present-day BSE [ε^53^Cr_BSE_ = 0.04 ± 0.02 (*12*)] reflects the ε^53^Cr composition of the hypothetical present-day PEM and Theia, mixed during Earth-Moon formation in the first 70 Ma of the Solar System, indicate that the building material for PE fractionated from the bulk Solar System no later than ~3 Ma after Solar System formation. The timing of PE reservoir formation is shifted toward older ages with increasing Mn/Cr differences between PE and Theia (Θ_Mn/Cr_).

The early Mn-Cr fractionation of PE, a planetary body depleted in volatile elements relative to the bulk solar composition, suggests that it accreted from volatile-poor materials or lost large amounts of its volatile budget during its accretion. The latter scenario could have been facilitated by magma ocean degassing or evaporation of rock due to energetic collisions between planetary bodies (*53*, *54*). Although the 1% evaporation temperature of Mn and Cr exceeds their 50% condensation temperature (*6*, *8*), these evaporation temperatures are not substantially higher than the liquidus temperatures of peridotite, which may have been commonly reached during large-scale melting events. Volatile loss from a planetary body also requires extensive convection during a magma ocean stage. Thus, volatile loss depends not only on the peak temperature but also on the duration it is sustained and the dynamics of the body. Young *et al.* (*55*) demonstrated that prolonged high-temperature conditions can cause ~10% losses of Si and Mg (elements more refractory than Mn and Cr), which could have caused pronounced Mn-Cr fractionation during these episodes. Nevertheless, it is expected from the ε^53^Cr models of PE that Mn/Cr depletion occurred early on, with minimal Mn-Cr fractionation from the PE reservoir after ~3 Ma of Solar System evolution, e.g., during the Earth-Moon forming impact event ≲70 Ma after CAI formation. Consequently, the Earth-Moon forming impact event may not have resulted in pronounced Mn-Cr fractionation during the vaporization processes of silicate melts. High escape velocities and the brief duration of extreme thermal conditions likely limited Mn and Cr loss from the Earth-Moon system. However, early collisions between small planetary bodies during the first few million years of the Solar System may have enabled Mn-Cr fractionation. The Mn-Cr fractionation ages of volatile element depleted CH and CB chondrites (that have Mn/Cr lower than any other chondrite group) suggest that impact-related chondrule formation involved sub-chondritic precursor bodies or impact-induced Mn-Cr fractionation by vaporization from silicate melts. The latter process could have fractionated Mn relative to Cr but likely operated only during small body impacts where the collision exceeded the escape velocity required for chemical elements to leave the target or impactor.

In contrast to CH and CB chondrites, CM, CO, CV, and CK chondrites exhibit step-function volatile element depletion patterns (*14*), which can be reconciled with nebula evaporation/condensation models (*15*, *56*, *57*). This suggests that nebula evaporation/condensation was likely the dominant process responsible for their volatile element inventory, reflecting the separation of gas from solids. Physical processes that decouple gas from solids are (i) the viscous evolution and associated gas drag in the protoplanetary disk, (ii) photo-evaporation of gas by high-energy photons (extreme ultraviolet photons, far ultraviolet photons, and x-rays) on the disk surface, and (iii) planet formation [e.g., (*1*)]. The reservoir fractionation ages of CCs and the PE may be related to the cessation of Mn-Cr fractionation by evaporation/condensation of nebula solids and gas due to the spatial dissipation of the disk. As most of the mass in the Solar System may also have condensed before this event, the cessation of Mn-Cr fractionation is not related to a boundary temperature but rather reflects limited solid-gas interaction within the disk. The disk region that formed the PE dissipated no later than ~3 Ma after CAI formation, similarly to disk regions at greater heliocentric distances, e.g. CC reservoirs. Consequently, it is expected that the inner Solar System planets Venus and Mercury accreted and differentiated similarly to the PE during the first few million years of the Solar System. Moreover, the Mn-Cr fractionation in the PE and meteoritic reservoirs (except CH and CB) within the first ~3 Ma of the Solar System provides a lower limit for the lifetime of the protoplanetary disk and transition to the debris disk stage. This agrees with astronomical constraints on protoplanetary disk lifetimes (*2*–*4*).

Meier *et al.* (*25*) showed that Giant Impact models assuming high angular momentum (*22*, *58*) can result in large-scale homogenization of isotope compositions during Earth-Moon formation, consistent with the nearly identical isotope compositions of the BSE and the Moon for many isotope systems. However, if the Earth and Moon inherited their distinct FeO contents from PE and Theia, respectively, then only Giant Impact models via “hit-and-run” (*59*), where PE and Theia had nearly identical compositions similar to enstatite meteorites, can account for this difference (*25*). This contrasts with a CC-like source for Theia. Giant Impact models, assuming high angular momentum (*22*, *58*), allow any known Solar System material for Theia. If material similar to CCs made up most of Theia, then only high angular momentum models are possible (*25*). These models align well with the chemical composition and many isotope signatures of the BSE, associated with a relatively small Theia (~0.1 M_E_) with a CI chondrite-like composition. While the high angular momentum “merger” model of Canup (*22*) implies a relatively large Theia (~0.4 M_E_) and requires that PE and Theia had more similar compositions, the formation of the Earth-Moon system via “impact-fission” (*58*) allows for a relatively small Theia with CI chondrite-like composition, supported by multiple chemical and isotopic evidence (*18*, *20*, *21*, *29*, *30*). The different chemical compositions and isotope signatures of PE and Theia suggest that they formed at different disk radii in the early Solar System. Consequently, Earth-Moon formation, where Theia had a CI chondrite-like composition, requires migration of Theia toward PE before the Giant Impact. This late delivery of volatile-rich material to Earth requires a major instability in the planetary arrangement of the early system that enabled the migration of a Mars-sized body from a region beyond Mars into the inner Solar System, where PE had formed. This delivery of relatively volatile-rich material to a strongly volatile-depleted PE may have been a turning point in Earth’s history, bringing with it the essential chemical elements in sufficient abundance for Earth to become a habitable planet on which life could emerge.

## MATERIALS AND METHODS

### Constraints on Solar System initial ^53^Mn/^55^Mn, ε^53^Cr, and the half-life of ^53^Mn

**Fig. 4.**
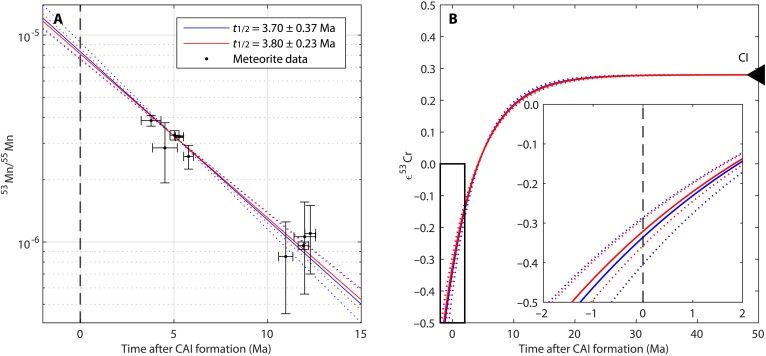
Evolution of bulk Solar System ^53^Mn/^55^Mn and ε^53^Cr with time. The evolution paths are modeled for different estimates for the half-life of ^53^Mn (*5*, *60*). (**A**) ^53^Mn/^55^Mn evolution lines are anchored to the D’Orbigny angrite with ^53^Mn/^55^Mn from (*62*) and a U-corrected Pb-Pb age from (*61*). Meteorite data of achondrites compiled in (*5*) are shown for comparison. (**B**) ε^53^Cr evolution curves are modeled by back-calculation from the present-day ^55^Mn/^52^Cr and ε^53^Cr of CI chondrite from (*12*).

Precise and accurate values for the half-life of ^53^Mn and the initial Solar System values of ^53^Mn/^55^Mn and ε^53^Cr are critical for establishing a robust timeline for early Solar System processes based on the ^53^Mn-^53^Cr chronometer. The linear correlation between the absolute ages of achondrites (Pb-Pb ages) and their ^53^Mn/^55^Mn (Fig. 4A) suggests that ^53^Mn was initially homogeneously distributed in the Solar System [e.g., (*5*)]. The correlation line constrains ^53^Mn/^55^Mn at any absolute point in time, while its slope constrains the half-life of ^53^Mn. Using this approach, Desch *et al.* (*5*) inferred the half-life of ^53^Mn to be 3.80 ± 0.23 Ma. This value is consistent with the independently derived half-life of ^53^Mn of 3.7 ± 0.4 Ma by Honda and Imamura (*60*), who used the specific activity of ^53^Mn from neutron activation by α-bombardment of a Cr target. Assuming that ^53^Mn/^55^Mn at 4563.51 Ma (*61*) was 3.24 × 10^−6^ (*62*), one can calculate the ^53^Mn/^55^Mn at any point in time (e.g., Solar System initial) with

(Mn53/Mn55)t=(Mn53/Mn55)DOeλ53(t−tDO) (1)

and the angrite D’Orbigny (DO) as the anchor for ^53^Mn/^55^Mn, whose absolute age *t*_DO_ [4563.51 ± 0.18 Ma (*61*)] and ^53^Mn/^55^Mn_DO_ [= 3.24 ± 0.04 × 10^−6^ (*62*)] are precisely constrained. Anchoring to DO angrite has generally been established as a robust approach in cosmochronometry, providing reliable and concordant ages between different short-lived chronometers (e.g., ^53^Mn-^53^Cr and ^182^Hf-^182^W). We note that the results do not change significantly when using the parameters of Desch *et al.* (*5*). These authors established a method without the need for an anchor by using a goodness-of-fit approach to meteorite data that depends on the choice of meteorite data. Taking an absolute age difference between the initial Solar System and isotopic closure in DO angrite of 5.03 ± 0.05 Ma (*5*), the age of the Solar System is 4568.54 ± 0.19 Ma. Small differences to the inferred initial of the Solar System by Desch *et al.* (*63*) (= 4568.42 ± 0.24 Ma) occur because these authors used an average Pb-Pb age for DO angrite of 4563.24 ± 0.21 [constrained with data from (*61*, *64*, *65*)], which includes U-uncorrected Pb-Pb ages, while this study only uses the U-corrected Pb-Pb age from Tissot *et al.* (*61*). The corresponding ^53^Mn/^55^Mn of the Solar System at 4568.54 Ma is $8.11_{-0.41}^{+0.49}$ × 10^−6^ with a half-life of ^53^Mn = 3.80 ± 0.23 Ma (*5*) or $8.31_{-0.68}^{+0.92}$ × 10^−6^ taking 3.70 ± 0.37 Ma (*60*) as the half-life.

Some of these parameters differ from previous studies that constructed progressive ε^53^Cr evolution models, assuming initial Solar System ^53^Mn/^55^Mn and ε^53^Cr inferred from bulk chondrite isochrons (*36*, *38*, *39*). Previous studies derived ε^53^Cr evolution curves that lacked consistency to a certain degree to respective present-day ε^53^Cr compositions of chondrites (*44*). This discrepancy is probably related to the assumption that the initial ε^53^Cr and ^53^Mn/^55^Mn of the Solar System can be derived from bulk chondrite isochrons (*11*, *66*), which, however, show no chronological significance (*12*). Moreover, the ε^53^Cr evolution model applied in the present study is related to an absolute timescale and thus independent of initial ε^53^Cr and ^53^Mn/^55^Mn at the absolute age of the Solar System, which are consensus of recent debate [e.g., (*63*)].

With these constraints, one can obtain the ε^53^Cr of a reservoir (*R*) at any absolute time by back-calculation from its present-day ε^53^Cr and ^55^Mn/^52^Cr

(Cr53/Cr52)t=(Cr53/Cr52)R−(Mn55/Cr52)R(Mn53/Mn55)DOeλ53(t−tDO) (2)

with

εCrR53=[(Cr53/Cr52)R(Cr53/Cr52)Std−1]×10,000 (3)

where (^53^Cr/^52^Cr)*_R_* and (^55^Mn/^52^Cr)*_R_* are the present-day ratios of the reservoir; (^53^Cr/^52^Cr)_Std_ is the terrestrial ratio [= 0.113386 (*67*)]; (^53^Mn/^55^Mn)_DO_ is the value of the angrite D’Orbigny [= 3.24 ± 0.04 × 10^−6^ (*62*)]; λ_53_ is the decay constant of ^53^Mn, where the half-life of ^53^Mn is 3.80 ± 0.23 Ma (*5*); and *t* and *t*_DO_ are the absolute ages corresponding to the time step and the age of D’Orbigny, respectively. Using these parameters, one can derive model ages for early Solar System processes and events that led to Mn/Cr fractionation.

CI chondrites have ε^53^Cr_CI_ = 0.28 ± 0.01 and (^55^Mn/^52^Cr)_CI_ = 0.84 ± 0.04 (*12*), which are the highest values among any chondrite group. They are chemically close to the Solar photosphere (*7*), thus representative of the bulk Solar System. Hence, back-calculating from the present-day ε^53^Cr and ^55^Mn/^52^Cr of CI chondrite constrains the initial ε^53^Cr of the Solar System, assuming that ^55^Mn was initially homogeneously distributed, and the Solar System had a unique initial ε^53^Cr. Figure 4B models the bulk Solar System evolution of ε^53^Cr by back-calculation from the present-day ε^53^Cr composition and ^55^Mn/^52^Cr of CI chondrite (representative of the bulk Solar System), which yields an initial ε^53^Cr value for the Solar System of −0.34 ± 0.09 with the half-life of ^53^Mn from Honda and Imamura (*60*) and −0.32 ± 0.06 with the half-life from Desch *et al.* (*5*). Magmatic iron meteorites, remnants of metal from the cores of the oldest known differentiated bodies, provide an upper limit for the initial Solar System ε^53^Cr of −0.272 ± 0.027 (*38*). Assuming that the half-life of ^53^Mn is at least 3.57 Ma (*5*), the ε^53^Cr evolution model (Fig. 4B) suggests that the Solar System must have been older than 4567.8 Ma. This absolute age is consistent with recent estimates of Solar System formation older than 4568 Ma [4568.22 ± 0.17 Ma (*68*); 4568.42 ± 0.24 Ma (*63*); and 4568.36 ± 0.59 Ma (*69*)], which contrasts with younger ages [e.g., 4567.30 ± 0.16 Ma (*70*)] due to possible resetting of CAIs (*63*). Assuming any other material as a representative of the bulk Solar System (ordinary or CC groups with depleted Mn/Cr and present ε^53^Cr relative to CI chondrite) would result in different ε^53^Cr evolutionary pathways of the Solar System, as discussed in Kruttasch *et al*. (*44*).

### Model calculations of PE reservoirs’ ^55^Mn/^52^Cr and ε^53^Cr through time

We calculated the ^55^Mn/^52^Cr of the PEM, PEC, and PE for three different mixing scenarios between PEM and Theia, resulting in the present-day ^55^Mn/^52^Cr and ε^53^Cr of the BSE. In model I, PEM and Theia had identical compositions similar to the BSE; in model II, 90% PEM and 10% Theia (± 5%) are mixed; and in model III 60% PEM and 40% Theia (± 5%) are mixed, where Theia has CI chondrite-like Mn/Cr, respectively. The mass-balance equations for the PE reservoirs in all scenarios are

(Mn55/Cr52)PEM=(Mn55/Cr52)BSE−(Mn55/Cr52)TΧT(1−ΧT) (4)

(Mn55/Cr52)PEC=(Mn55/Cr52)PEMDMn/Crm−s (5)

(Mn55/Cr52)PE=(Mn55/Cr52)PEM(1−ΧPEC)+(Mn55/Cr52)PECΧPEC (6)

where ^55^Mn/^52^Cr_BSE_ and ^55^Mn/^52^Cr_T_ are the ^55^Mn/^52^Cr of the BSE and Theia (T), respectively, and Χ_T_ is the bulk mass fraction of Theia. Models II and III assume that ^55^Mn/^52^Cr_BSE_ = 0.475 (*13*) and ^55^Mn/^52^Cr_T_ = ^55^Mn/^52^Cr_CI_ [= 0.84 (*12*)], while model I assumes ^55^Mn/^52^Cr_T_ = ^55^Mn/^52^Cr_BSE_. The ^55^Mn/^52^Cr_PEC_ is calculated for a range of metal-silicate partition coefficients of Mn relative to Cr, where $D_{\text{Mn}/\text{Cr}}^{m-s}=D_{\text{Mn}}^{m-s}/D_{\text{Cr}}^{m-s}<0.16$ (*9*, *10*). Both elements fractionate into metal relative to silicate with increasing temperature, decreasing oxygen fugacity, and decreasing S and C concentrations in metal, while changes in pressure have little effect (*9*). In addition, the affinity of Cr for metal is stronger than that of Mn, leading to $D_{\text{Mn}}^{m-s}/D_{\text{Cr}}^{m-s}$ < 0.16 for less than ~20 wt % S in metal (*9*, *10*), which is higher than the expected S content in Earth’s core, i.e., ~1.7 wt % S (*71*). The mass fraction of Earth’s core (Χ_PEC_) is 0.325 (*72*).

The coefficient Θ_Mn/Cr_ describes the differences in ^55^Mn/^52^Cr between PEM and Theia in the three different mixing scenarios and is calculated from

ΘMn/Cr=∣(Mn55/Cr52)T−(Mn55/Cr52)PEM∣(Mn55/Cr52)BSE (7)

The hypothetical present-day ε^53^Cr compositions of PEM, PEC, and PE are

εCrPEM53=εCrBSE53−εCrT53χT(1−χT) (8)

εCr0CI53≤εCrPEC53≤εCrPEM53 (9)

εCrPE53=εCrPEM53(1−χPEC)+εCrPEC53χPEC (10)

where ε^53^Cr_BSE_ and ε^53^Cr_T_ are the values of the BSE and Theia, with ε^53^Cr_BSE_ = 0.04 ± 0.02 (*12*) and ε^53^Cr_T_ = ε^53^Cr_CI_ [= 0.28 ± 0.01 (*12*); model II and model III] or ε^53^Cr_T_ = ε^53^Cr_BSE_ (= 0.04 ± 0.02; model I). We note that the Cr concentration of the BSE [= 2650 μg/g (*13*)] is close to that of CI chondrite [= 2720 μg/g (*14*)], and this difference is smaller than the estimated uncertainty of the Cr mass fraction in Theia (χ_T_) of ±5%. The Cr mass fraction in PEC is similar to that of the BSE as the partioning of Cr between metal-silicate is approximately equal. The ε^53^Cr_PEC_ lies between the initial ε^53^Cr of the Solar System and the present-day ε^53^Cr of PEM. The mass fractions of Cr in Theia and PEC ( χ_T_ and χ_PEC_) correspond to Χ_T_ and Χ_PEC_ in Eqs. 4 and 6.

Monte Carlo simulations (*n* = 10,000) constrain the ε^53^Cr evolution of PE-PEM using variable ^55^Mn/^52^Cr of PE and PEM, present-day ε^53^Cr of PE (Table 2), and core formation times (*t*_1_) between 0 and 70 Ma. The ε^53^Cr evolution of PE-PEM applies Eq. 11 from *t*_0_ to *t*_1_, and Eq. 13 from *t*_1_ to the present-day value

(Cr53/Cr52)PEt=(Cr53/Cr52)PE0+(Mn55/Cr52)PE(Mn53/Mn55)O(1−e−λ53t) (11)

with

(Cr53/Cr52)PE0=(Cr53/Cr52)PE−(Mn55/Cr52)PE(Mn53/Mn55)O(1−e−λ53t0) (12)

and

(Cr53/Cr52)PEMt=(Cr53/Cr52)PEt1+(Mn55/Cr52)PEM(Mn53/Mn55)t11−e−λ53(t−t1) (13)

Reservoir formation ages result from solving the intersection between their evolution curves with that of the bulk Solar System (CI) using

ΔTX−CAI=1λ53ln[(Mn53/Mn55)0(Mn53/Mn55)X] (14)

and

(Mn53/Mn55)X=(Cr53/Cr52)R−(Cr53/Cr52)CI(Mn55/Cr52)R−(Mn55/Cr52)CI (15)

where (^53^Mn/^55^Mn)_0_ and (^53^Mn/^55^Mn)*_X_* are the ratios at the beginning of the Solar System (at 4568.54 Ma) and fractionation from the bulk Solar System, respectively. The abbreviation *R* refers to the reservoir fractionated from the bulk Solar System (CI) reservoir.
